# Health-related quality of life in the Brazilian Amazon: a population-based cross-sectional study

**DOI:** 10.1186/s12955-017-0734-5

**Published:** 2017-08-14

**Authors:** Marcus Tolentino Silva, Monica Caicedo Roa, Tais Freire Galvao

**Affiliations:** 1 Faculty of Medicine, Federal University of Amazonas, Rua Afonso Pena, 1053, Manaus, Amazonas CEP: 69020–160 Brazil; 2 Faculty of Medicine, Clinical Research Institute, National University of Colombia, Calle 30 No. 45–03 Ciudad Universitaria, Bogotá, Colombia; 3 Faculty of Pharmaceutical Sciences, University of Campinas, Rua Cândido Portinari, 200, Cidade Universitária, Campinas, São Paulo CEP: 13083–871 Brazil

**Keywords:** Quality of life, Health behavior, Health care surveys, Patient preference, health status, Brazil

## Abstract

**Background:**

To analyze perceptions of health-related quality of life and associated factors in populations from the Manaus Metropolitan Region.

**Methods:**

We conducted a population-based cross-sectional study from May to August 2015. Adults aged 18 years and older were selected using probabilistic three-phase cluster sampling and stratified by sex and age, based on official estimates. Quality of life data were collected using the European Quality of Life 5-Dimensions 3-Levels (EQ-5D-3L) along with socioeconomic, demographic, and health perception data. Utility scores were calculated using the Brazilian version of the EQ-5D-3L. Descriptive statistics were derived, and a multivariate Tobit regression model with correction for complex sampling was performed to identify the variables that influence utility levels.

**Results:**

A total of 4001 participants were included. The average utility score was 0.886 (95% confidence interval [CI]: 0.881–0.890) with significant differences according to living area (the capital (0.882 ± 0.144) or inner cities (0.908 ± 0.122; *p* < 0.001)). The dimension for which the highest proportion of people reported moderate to severe problems was pain/discomfort (39%), followed by anxiety/depression (18%). Men had a higher quality of life than women (β = 0.041, *p* < 0.001). Not working was a factor that increased quality of life compared with being formally employed (β = 0.031, *p* = 0.037). The poorest people had a lower quality of life than the richest people (β = −0.118, *p* < 0.001). Better health perceptions increased utility scores (*p* < 0.001), while being separated decreased the scores (β = −0.052, *p* = 0.001).

**Conclusion:**

Health-related quality of life in the Manaus Metropolitan Region was high, as expected for the general population, and was higher among individuals who lived in the inner cities, men and those in higher social classes. Gender discrepancies and differences in quality of life between the capital and inner cities should be further investigated.

## Background

Quality of life is a broad, multidimensional concept that includes a subjective assessment of positive and negative life aspects [1]. It is a popular term that encompasses the physical, mental and social dimensions of well-being. Aspects such as culture, values and spirituality are also essential components of quality of life and contribute to its complexity [2].

Measures of health-related quality of life refer to the perceptions of an individual or group of individuals of their physical and mental health over time [2]. This estimate is widely accepted as a relevant outcome, and it has been applied to assess needs, measure the burden of disease and disability, guide the use of health resources and assess progress toward achieving health goals [2, 3].

Monitoring and analyzing health-related quality of life helps to identify population groups with low perceived quality of life and allows these groups to be prioritized when public health policies are implemented to address risk factors. Additionally, this outcome is relevant in clinical and economic studies [3, 4].

Several instruments have been proposed to measure health-related quality of life, including the European Quality of Life (EuroQol) 5-Dimensions (EQ-5D), a generic self-report tool developed by the EuroQol group in 1991 to measure preferences [5]. It has been widely applied in population studies in Europe [6, 7], Latin America [8, 9], China [10], Australia [11, 12] and other countries [13–15]. Recently, its utility scores were validated for the Brazilian population [13], but studies on the quality of life of Brazilians are still scarce.

Manaus is the capital of Amazonas, the largest state in Brazil and the municipality with the sixth-highest percentage of gross domestic product in the country [16]. The main causes of morbidity and mortality are violence, mainly deaths by firearms [17], communicable diseases (tuberculosis, leprosy and HIV/AIDS) and vector-borne diseases (malaria and dengue) [18]. In addition, chronic and neoplastic diseases influenced by environmental factors play an important role [19]. Assessments of the quality of life of the people living in this region remain absent so far. Such appraisals would enable the measurement of the population’s health status and allow an understanding of the factors that affect this outcome.

The aim of this study was to analyze the health-related quality of life and associated factors in the population of the Manaus Metropolitan Region through the application of EQ-5D in a large representative survey.

## Methods

### Study design

From May to August 2015, we conducted a cross-sectional, population-based study in the Manaus Metropolitan Region, which includes the capital and seven other municipalities of the state of Amazonas: Careiro da Várzea, Iranduba, Itacoatiara, Manacapuru, Novo Airão, Presidente Figueiredo, and Rio Preto da Eva. The study protocol is available for further information.

### Setting

Manaus is the capital of Amazonas. Since the 1960s, it has witnessed intense migratory flows that have led to unplanned urban sprawl.

Over two million inhabitans live in Manaus Metropolitan Region, more than 60% of the Amazonas state population [31]. Its main economic activities include the household appliances industry, trade and tourism. Access from other Brazilian areas to these cities is precarious and is available only by river or air. In 2010, the human development index of the city of Manaus was 0.737; for the metropolitan region, it was 0.720 [20]. The present analysis is part of a larger study examining the use of health services and inputs in this region.

### Sample size

To calculate the sample size, we considered the official estimates of the population and an estimated health services use of 50%. We considered an α level of 0.05, power of 20%, and a design effect of 1.5. A sample size of 3598 people was calculated with a confidence level of 95%. An additional 10% of participants were included to compensate for potential losses.

### Participants

Adults aged 18 years old and older were eligible for the study. A probabilistic three-phase cluster sample was performed for the 2647 urban sectors of the Manaus Metropolitan Region. We randomly selected 400 primary sectors and 20 replacements (first phase) in each sector. To select households, we applied systematic sampling (second phase). Finally, we randomly selected individuals from the residences according to predefined quotas of sex and age, based on census tracts’ official estimates (third phase).

### Variables, data collection and measurement

The primary outcome was the health-related quality of life, as measured with the mean utility score. For simplicity, “utility score” or “score” are used to refer to “mean utility score”. When interpreting the results, such scores are referred to simply as “quality of life”.

Data collection was conducted through semi-structured standardized interviews by staff who were previously trained in quantitative research. Social, demographic and economic income data were collected. Detailed information can be found in the previously published protocol.

Socioeconomic data were collected according to the five strata (A to E) of the Brazilian economic classification criteria, where A signifies wealthier status and E signifies poorer status [21]. This criterion allows an estimation of the household monthly income in Brazilian real (BRL), which was converted to US dollars (USD) based on the currency of the Central Bank of Brazil on July 1, 2015: 1 USD = 3.1185 BRL).

Self-perceptions of health status were measured with the question “*In general, what is your health status?”* (possible answers: very good, good, fair, bad, very bad).

Perceptions of quality of life were assessed by applying the 3-level version of the EQ-5D (EQ-5D-3L) in its validated Brazilian Portuguese version [22]. The EQ-5D-3L includes five dimensions with questions regarding physical function and disability (mobility, self-care, pain/discomfort), mental function (anxiety/depression) and social activity (usual activities) [3]. Each dimension includes three levels of responses: 1 = no problems, 2 = moderate problems and 3 = severe problems.

The three levels of responses on the EQ-5D-3L are categorized using one number from 1 to 3 that expresses the level selected for each dimension, and these numbers are then combined to create five numbers representing the respondent’s state of health. These combinations range from 11111 (no problems in any dimension) to 33333 (severe problems in all dimensions), allowing the expression of 243 health states [4]. For instance, state 11123 would indicate no problems with mobility, self-care, and usual activities, moderate problems with pain/discomfort, and severe problems with anxiety/depression.

Utility scores were obtained from a validation study that included 9148 people in the state of Minas Gerais and the cities of Rio de Janeiro, Porto Alegre, and Recife [23]. To transform the five-digit health status to health utility values, we used the following formula [23]:

Utility score = 0.851+ (−0.120*M2) + (−0.363*M3) + (−0.112*SC2) + (−0.218*SC3) + (−0.097*UA2) + (−0.184*UA3) + (−0.064*PD2) + (−0.168 *PD3) + (−0.050*AD2) + (−0.095*AD3).

**M**: mobility; **SC**: Self-care; **UA**: Usual activities; **PD**: Pain and discomfort; **AD**: Anxiety/depression; 2, moderate problems in the dimension; 3, severe problems in the dimension.

### Statistical analysis

We used descriptive statistics for the demographic and socioeconomic qualitative variables of the population, with estimates of absolute and relative frequencies. For the utility scores, the mean, standard deviation and 95% confidence interval (CI) were calculated.

A multivariate Tobit regression model with limits of 0 and 1 was performed to assess the socioeconomic and demographic variables. This model was chosen considering the facts that the utility variable is censored, is not normally distributed and has ceiling effects. A traditional regression model with least squares estimation was not employed since the coefficients of this analysis could be biased by the true population parameters regardless of sample size [24].

Unavailable data were replaced with missing values for the Tobit models, and no imputations were performed. All variables were included in the adjusted model, which was corrected for the complex sampling method. The analysis was stratified by the total sample, the capital of Manaus and the inner cities. A separate model for women who had been pregnant in the last 12 months was conducted to determine the impact of maternity on quality of life. For the data analysis, we used the statistical program Stata 14.2 [25].

## Results

We invited 5410 people, of whom 1409 refused to participate or were found ineligible. A sample size of 4001 participants was included. Of the evaluated sample, 87% resided in the capital, and the remaining 522 lived in the inner cities: Careiro da Várzea (41), Iranduba (70), Itacoatiara (134), Manacapuru (140), Novo Airão (20), Presidente Figueiredo (57) and Rio Preto da Eva (40).

The general population characteristics are shown in Table 1. Both sexes were distributed similarly in the sample, with higher scores for men (unadjusted *p* < 0.001). The main age group represented was from 25 to 34 years old (28.8%); utility scores were lower in the higher age groups (unadjusted *p* ≤ 0.004). People who identified their race as brown (mixed-race) were predominant (72.2%). The 1% of participants who identified themselves as indigenous reported the lowest utility scores among the ethnic categories; however, the difference was not significant (0.863; unadjusted *p* = 0.148).

**Table 1:** Demographic characteristics of the participants (*n* = 4001) and mean utility scores

Variables	n	%^a^	Mean utility scores	*p* value^c^
Sex				
Female	2113	52.8	0.872	Ref.^d^
Male	1888	47.2	0.901	<0.001
Age group (years)				
18–24	838	20.9	0.912	Ref.
25–34	1152	28.8	0.904	0.174
35–44	843	21.1	0.891	0.004
45–59	772	19.3	0.858	<0.001
≤60	396	9.9	0.822	<0.001
Marital status				
Single	2173	54.3	0.898	Ref.
Separated/divorced	260	6.5	0.843	<0.001
Widowed	159	4.0	0.825	<0.001
Married	1409	35.2	0.881	<0.001
Pregnancy (last 12 months)				
No	1890	89.5	0.870	Ref.
Yes	223	10.5	0.891	0.051
Ethnicity				
White	636	15.9	0.897	Ref.
Black	300	7.5	0.904	0.196
Asian	138	3.5	0.881	0.265
Brown	2886	72.2	0.882	0.012
Indigenous	41	1.0	0.863	0.148
Educational level				
Higher education or above	158	4.0	0.892	Ref.
High school	1903	47.5	0.903	0.300
Middle school	649	16.2	0.899	0.543
Elementary school or less	1291	32.3	0.853	0.004
Work status				
Formal	761	19.0	0.907	Ref.
Informal	1149	28.8	0.882	<0.001
Retired	315	7.9	0.808	<0.001
Unemployed	1199	29.9	0.890	0.034
Does not work	577	14.4	0.899	0.957
Social class^e^				
A/B1	124	3.1	0.930	Ref.
B2	505	12.6	0.921	0.456
C1	862	21.5	0.905	0.032
C2	1423	35.6	0.884	<0.001
D/E	1087	27.1	0.852	<0.001
Health perception				
Very good	471	11.9	0.920	Ref.
Good	2175	54.3	0.921	0.514
Fair	1108	27.7	0.843	<0.001
Bad	193	4.8	0.738	<0.001
Very bad	54	1.3	0.607	<0.001

^a^Frequency adjusted by sample complex design

^b^HRQoL values were measured by European Quality of Life 5-Dimensions 3-Levels instrument and transformed as utility values based on values from a previous study in the Brazilian population [28]

^c^Tobit unadjusted regression analysis

^d^Ref., reference

^e^Social class according to the Brazilian criteria of economic classification [26]

Half of the sample (47.5%) had completed their education through high school; this group presented the highest utility scores (0.903) among the education attainment strata, and those with incomplete educational levels showed lower levels of quality of life (unadjusted *p* = 0.004). Eight-four percent belonged to socio-economic classes C, D and E. The higher social classes (A and B1, with average monthly household incomes of USD 2788–6500) represented 3.1% of the population and had a utility score of 0.930, which differed significantly from the utility scores of the lower economic classes C2 (0.884) and D/E (0.852; unadjusted *p* < 0.001). Formally employed participants represented 19% of the sample and had higher scores (0.907) that differed significantly from the scores of those who were retired or informally employed (unadjusted *p* < 0.001). Most participants (66.2%) identified themselves as being in good or very good health with a utility score of 0.920, which was significantly higher than the percentage that self-identified as having fair, good, or very bad health (unadjusted *p* < 0.001).

**Fig. 1:**
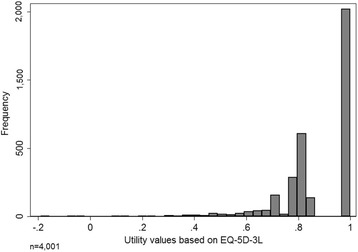
Distribution of utility scores in the population of Manaus Metropolitan Region, Brazil based on the European Quality of Life 5-Dimensions 3-Levels (EQ-5D-3 L)

The mean utility score of the study population was 0.886 (95% CI: 0.881–0.890), range: −0.189 to 1. Figure 1 shows the left-skewed distribution (skewness = −1.48). Half of the participants reported a state of 11111 (no problems in any dimension; 52.6%). It was possible to identify health states with negative values, representing states worse than death. In the study population, five people rated their quality of life negatively, with health-related quality of life scores ranging from −0.026 (33322) to −0.889 (33331).

**Fig. 2:**
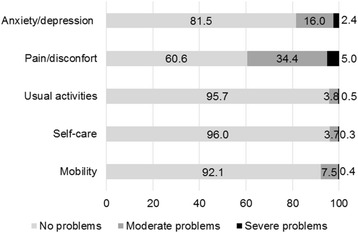
Self-perceived health of the population of Manaus Metropolitan Region, Brazil according to European Quality of Life 5-Dimensions 3-Levels

The dimension for which the highest proportion of people reported moderate to severe problems was pain/discomfort, followed by anxiety/depression (Fig. 2). Moderate problems with pain/discomfort (state 11121) were reported by 20.2%, and severe problems in this dimension (11131) were reported by 2.12%; 4.89% reported moderate or severe problems in the anxiety/depression dimension, and two people were rated 33333.

**Fig. 3:**
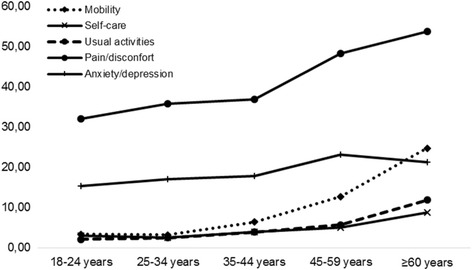
Proportion of people with moderate and severe problems in the dimensions of European Quality of Life 5-Dimensions 3-Levels by age group

One-third of the young adults (18 to 24 years old) reported some problems in the pain/discomfort dimension, and 15.4% reported problems with anxiety/depression. This proportion increased in each age group, with a higher proportion of moderate and severe problems in each dimension among people aged 60 years and older (Fig. 3).

The utility scores of individuals from the capital (0.882 ± 0.144) were lower than those of people from the interior (0.908 ± 0.122, *p* < 0.001). We identified 90 health states in the population out of the 243 possibilities using the EQ-5D-3L. In Manaus, 87 health states were reported, while in the inner cities, 25 health states were reported.

The adjusted models showed that men had higher utility scores than women (β = 0.041, *p* < 0.001). This difference was also observed in analyses of the capital (β = 0.038, *p* < 0.001) and the inner cities (β = 0.057, *p* = 0.028; Table 2). Separated/divorced people had a lower quality of life than single people (β = −0.052, *p* < 0.001); this difference was significant in the capital (β = −0.046, *p* = 0.008) but not in other cities in the area.

**Table 2:** Adjusted impact of study variables on utility scores by Tobit regression models of total sample, the capital and and inner cities

Variables	Total (*n* = 4001)		Manaus (*n* = 3479)		Inner cities (*n* = 522)	
	Coefficient	*p* value	Coefficient	*p* value	Coefficient	*p* value
Sex						
Female	Ref.^a^		Ref.		Ref.	
Male	0.041	<0.001	0.038	<0.001	0.057	0.026
Age group (years)						
18–24	Ref.		Ref.		Ref.	
25–34	−0.002	0.849	−0.005	0.692	0.006	0.858
35–44	−0.004	0.787	0.001	0.972	−0.028	0.461
45–59	−0.028	0.053	−0.026	0.089	−0.054	0.164
60+	−0.022	0.270	−0.019	0.373	−0.072	0.187
Marital status						
Single	Ref.		Ref.		Ref.	
Separated/Divorced	−0.052	0.001	−0.046	0.008	−0.065	0.145
Widowed	0.012	0.614	0.015	0.578	0.023	0.748
Married	−0.006	0.485	−0.003	0.743	−0.008	0.752
Pregnancy (last 12 months)						
No	Ref.		Ref.		Ref.	
Yes	−0.005	0.789	−0.021	0.287	0.120	0.065
Ethnicity						
White	Ref.		Ref.		Ref.	
Black	0.036	0.066	0.014	0.516	0.148	0.005
Asian	0.006	0.791	0.013	0.589	−0.060	0.431
Brown	−0.019	0.095	−0.015	0.211	−0.029	0.329
Indigenous	−0.041	0.280	−0.057	0.160	0.011	0.900
Educational level						
Higher education or above	Ref.		Ref.		Ref.	
High school	0.029	0.181	0.023	0.329	0.043	0.423
Middle school	0.042	0.077	0.038	0.146	0.052	0.376
Elementary school or less	0.028	0.217	0.017	0.497	0.076	0.173
Work status						
Formal	Ref.		Ref.		Ref.	
Informal	−0.019	0.114	−0.019	0.134	−0.006	0.843
Retired	−0.042	0.056	−0.050	0.036	0.025	0.672
Unemployed	0.023	0.081	0.020	0.152	0.076	0.041
Does not work	0.031	0.037	0.029	0.067	0.069	0.100
Social class^b^						
A/B1	Ref.		Ref.		Ref.	
B2	−0.016	0.567	−0.010	0.732	−0.053	0.622
C1	−0.053	0.050	−0.043	0.133	−0.108	0.284
C2	−0.081	0.002	−0.078	0.005	−0.072	0.471
D/E	−0.118	<0.001	−0.103	<0.001	−0.204	0.041
Health perception						
Very good	Ref.		Ref.		Ref.	
Good	0.016	0.233	0.001	0.922	0.095	0.008
Fair	−0.115	<0.001	−0.133	<0.001	0.012	0.765
Bad	−0.242	<0.001	−0.252	<0.001	−0.169	0.005
Very bad	−0.369	<0.001	−0.379	<0.001	−0.299	<0.001

^a^Ref., reference

^b^Social class according to the Brazilian criteria of economic classification [23]

Not working, a category that included students and housewives, was a factor that increased utility scores compared with the formally employed in the whole sample (β = 0.031, *p* = 0.037), while being retired decreased the scores in the Manaus population (β = −0.050, *p* = 0.036) and being unemployed had the opposite effect among the population in the inner cities (β = 0.076, *p* = 0.041).

Social classes of C and lower were associated with significantly lower utility scores when compared to the wealthiest class. Belonging to classes D and E represented an adjusted mean reduction of −0.118 in utility scores for the total sample (*p* < 0.001); of −0.103 for the population in Manaus (*p* < 0.001); and of −0.204 for the population in the inner cities (*p* = 0.041).

Those with the poorest self-perceived health had significantly lower utility score in all models. Age, pregnancy, and education did not influence quality of life. With the exception of black people in the inner cities (β = 0.148, *p* = 0.005), race also did not affect the outcome.

## Discussion

Quality of life was higher in the inner cities than in the capital, Manaus. Being male, belonging to higher social classes, reporting better health status and not working increased quality of life. Being separated from a spouse negatively affected the outcome.

We chose the EQ-5D-3L due to its easy and quick application and its broad use in studies worldwide, which allowed for comparisons of populations within and between different countries [9, 26, 27]; however, as a generic instrument, its discriminant ability is lower than that of specific instruments [28, 29]. Another limitation of the present study was the lack of measurements of physical activity, fruit and vegetable consumption, religious beliefs, chronic diseases or leisure time, which are factors that can impact quality of life [30].

A quarter of the invited population refused to participate. Efforts to improve representativeness included the random selection of one subject per household using predefined quotas for sex and age based on the official estimates [31]. Refusal rates in epidemiologic studies are increasing in different contexts due to several factors, including individualism in the society [32].

The mean utility score was 0.886, representing the expected high quality of life for general population samples, which tend to be elevated [33, 34]. The most affected dimension was pain/discomfort, and the least affected dimensions were self-care and usual activities. These results were consistent with other population-based studies conducted in Brazil in the Federal District [13] and Minas Gerais [35] and in other countries, such as Poland [36], Germany [37], Australia [12] and China [26].

Half of the population mentioned having no problems in any of the dimensions (state 11111), which is expected in a representative sample in which most subjects are healthy. This proportion of healthy subjects was slightly higher than that found in previous studies conducted in Brazil (Minas Gerais, 44.3%) [35], UK (43%) [38] and Sweden (46%) [39]; however, it was lower than the proportion observed in studies conducted in China (87%) [14], Germany (66%) [37] and Spain (73%) [27].

We identified 90 health stages in the population, which was less than in other population-based studies [14, 15]; this result was probably related to sample size. A wider variation of states was observed in the capital than in the inner cities.

The quality of life of women was lower than that of men both in the general model and in the analyses stratified by city. These results were consistent with other studies, in which women reported lower quality of life than men [12, 13, 27, 40]. Factors associated with the lowest health quality in women have been described in terms of access to health services, formal education, physical mobility, sexual restrictions, segregation, less security and informal occupations [41, 42]. These and other factors need to be addressed from the gender perspective to reduce gaps in health equity.

In our population, quality of life decreased non-significantly with age. In previous studies, the elderly were the most affected group [12, 26, 30], and age was the most important socio-demographic variable that explained the lowest utility scores [43].

A controversial result found in our study was the higher quality of life of the unemployed in inner cities and of people who did not work for the whole sample. We ran additional analyses separating students from housewives, and the finding of significantly higher quality of life in people who were not working remained (β = 0.026, *p* = 0.035). Other variables’ regression results did not significantly change.

Income positively affected quality of life, while education attainment had no effect on this outcome. Economic resources can affect access to medical care, safer homes and neighborhoods, healthier food, leisure time and physical activity [44]. Higher income is associated to wellbeing and longevity [45]. Conversely, better educated people are more likely to have jobs with healthier working conditions, better health insurance, and higher wages [45].

As expected, higher health perception was associated with higher health-related quality of life. Despite the possible collinearity of independent and dependent variables, self-perception is a personal grade of health, while utility scores from EQ-5D led to a structured reflection of each dimension of the health state. Classic conceptual frameworks consider general health perception and health-related quality of life as distinct but related measures [46].

Quality of life is a measure that is increasingly being introduced as formal evidence to support the decision-making process in clinical and public health as well as economic evaluations for the allocation of health resources. Examples of this application are recommendations by the UK National Health Service [47] and New Zealand Pharmaceutical Management Agency [5]. In Brazil, the constitution of the Commission for Incorporating Technology in the Unified Health System is increasingly using cost utility analyses and social preferences to support the decision-making process [48]. In this context, heath-related quality of life studies provides an approximation of health perceptions and supports the development of strategies that positively impact the health of populations and reduce gaps between classes.

This was the first study performed in the Brazilian Amazon to evaluate quality of life in a large sample of the population with good representativeness. The utility scores were based on the Brazilian population [23], providing a more accurate measurement. The regression considered non-normal distributions and expected ceiling effects. The results may be useful for clinical and economic evaluations of health outcomes in Brazil.

## Conclusions

The health-related quality of life of people from Manaus Metropolitan Region is high, as expected for general population. Quality of life was higher among individuals who lived in the inner cities than among those who lived in the capital and was positively influenced by male gender and higher social class. In terms of policy, the present results highlight the need to reduce gender and social inequities in the Manaus Metropolitan Region. Pain and affective domains were the most affected. Gender discrepancies and differences in quality of life between those from the capital and those from inner cities should be further investigated.

## Abbreviations

AD: Anxiety/depression
BRL: Brazilian real
CI: Confidence interval
EQ-5D-3L: European quality of Life 5-dimensions 3-levels
HIV/AIDS: Human immunodeficiency virus/Acquired immune deficiency syndrome
PD: Pain and discomfort
SC: Self-care
UA: Usual activities
USD: United States dollars
